# Maternal effects and recessive epistasis govern green, yellow and brown seed coat color inheritance in soybean [*Glycine max* (L.) Merr.]

**DOI:** 10.1186/s40659-025-00648-9

**Published:** 2025-11-10

**Authors:** Rahul Kumar, Akshay Talukdar, Manisha Saini, Nenavath Krishna Kumar Rathod, Raju Ratan Yadav, Rohit Kumar Mahto, Renu Pandey, Kishor Gaikwad, S. K. Lal, Amitabha Bandyopadhyay

**Affiliations:** 1 ICAR Research Complex for N.E.H. Region, Tripura Centre, Lembucherra, India; 2 Division of Genetics, ICAR-Indian Agricultural Research Institute (IARI), New Delhi, India; 3 IC AR-Indian Institute of Sugarcane Research, Lucknow, India; 4 Department of Molecular Biology and Genetic Engineering, G.B. Pant, University of Agriculture and Technology, Pantnagar, Uttarakhand India; 5 School of Biotechnology, Institute of Science, Banaras Hindu University (BHU), Varanasi, Uttar Pradesh India; 6 Division of Plant Physiology, ICAR-Indian Agricultural Research Institute, New Delhi, India; 7 Division of Molecular Biology and Biotechnology, ICAR- National Institute for Plant Biotechnology, New Delhi, India; 8 Eden Flora-I, Kolkata, WB India

**Keywords:** Soybean, Seed coat color, Inheritance, Maternal effect, Gene interaction

## Abstract

Seed coat color is crucial for consumer preference in soybeans. This study explores the genetic mechanisms underlying yellow, green, and brown seed coats through reciprocal crosses, revealing that seed coat color is maternally inherited, with F_1_ seeds matching the female parental phenotype. In the F_2_ generation, all seeds had green coats, while F_3_ segregation patterns followed a two-gene epistatic model. The yellow (SKAF148) x brown (AGS457) cross segregated in a 9:3:4 ratio (green: yellow: brown), while yellow x green (SKAF148 x AGS346) segregated in a 3:1 ratio (green: yellow). Here, we report that two loci, *G1* and *G2*, govern color expression. Dominant alleles at both loci (*G1_G2_*) produced green seed coats, while yellow required *G1_g2g2* and brown required homozygous recessive *g1g1* alleles, demonstrating recessive epistasis where *g1g1* masked *G2* effects. This research establishes a genetic pathway from brown to yellow to green, offering key insights into the digenic inheritance of seed coat color. The parental genotypes were inferred as *G1G1g2g2* (yellow), *G1G1G2G2* (green), and *g1g1G2G2* (brown). These findings provide valuable guidance for breeding programs targeting consumer-preferred seed coat colors in soybeans.

## Key message

This study reveals that soybean seed coat color (yellow, green, brown) follows two-gene epistatic inheritance, offering insights in digenic inheritance to support breeding programs for desired colors.

## Introduction

Soybean [*Glycine max* (L.) Merr.] is the most widely grown grain legume in the world. Soybean contributes the major share of vegetable oil and plant-based proteins for mankind and domestic animals [9]. The crucial factors that affect seed yield and consumer acceptance of soybean includes amongst all the seed size, seed shape, and seed coat color [8, 26]. The color of the seed coat varies from black in the wild type soybean (*Glycine soja*) to a variety of colors viz., yellow, brown, green, or bicolor in the cultivated soybean (G max) [1, 10, 19]. However, the commercial cultivars of soybean primarily have yellow seed coat with a variety of hilum colors including brown, black, imperfect black, grey, and yellow [3, 18]. For ease of observation, the seed coat color has been in use as a phenotypic marker in soybean breeding [7, 20].

Studies to understand the genetic control of seed coat color development in soybean dates back to 1918 [21], however, the findings are contradictory indicating the complexity of the trait. So far, involvement of six genes viz., *I, R, T, W1, O,* and *G*, and their interactions have been reported to be responsible for the development of seed coat and hilum color in soybean [14, 27]. The genes *R,* and *T* are involved in the production of the pigments independently while genes *O* and *W1* is expressed only in presence of the combination of other two genes i.e., *i r* or *i t*, respectively. The recessive allele i contributes to seed color development in the seed coat while the dominant allele I inhibits it [2, 12, 25]. Two other alleles of *I* i.e. *i*^*i*^ and *i*^*k*^ allows development of color in the hilum and saddle areas of the seed coat. For the other color developing gene *R*, black pigment (*R_*) dominates over brown (*rr*) [12, 25]. The gene *T* exert its effect on the color development of the pubescence from dark brown (*TT*) through light brown (*Tt*) to grey (*tt*) [25]. Similarly, the gene *G* makes seed coat green (*G_*), which is dominant over yellow (*gg*) [22]. It is linked to one of the two complementary recessive nuclear genes (*d1*) that regulate chlorophyll retention throughout the seed [24, 25]. Reese et al. [17] reported another gene (*G2*) for green seed coat. Thus, the inheritance of seed coat color is yet to be fully elucidated. Therefore, an attempt was made to decipher the genetics of brown, green and yellow seed coat involving vegetable type (AGS457, AGS346) and seed type (SKAF148) soybeans. Seed coat being a maternal tissue, data from both forward and reciprocal crosses was involved in the study.

## Materials and methods

### Plant material

In this study, two vegetable soybean genotypes viz., AGS457 and AGS346 and one seed type soybean genotype i.e. SKAF148 were used. The seed coat of AGS 457 and AGS346 were brown and green, respectively while that of SKAF148 was yellow (Table 1). The seeds of the vegetable soybean genotypes were obtained from the World Vegetable Centre, Taiwan and the seeds of SKAF148 were obtained from the Sipani Krishi Anusandhan Farm (SKAF), Mandsaur, MP, India.

**Table 1:** Seed coat color and genotype of parentsand their F_1_ and F_2_s

CrossNo	Parents and cross combinations	Seed coat color of F_1_ seeds	Seed coat color of F_2_ seeds	Parentage or origin
	AGS 457 (Brown seed coat)	–	–	Dada Cha 2000 x [Dada Cha 2000 x (Taisho Shiroge x Neu Ta Pien 1)]
	SKAF148 (Yellow seed coat)	–	–	Sipani Krishi Anusandhan Farm, MP, India
	AGS 346 (Green seed coat)	–	–	[Ryokkoh x (Shih Shih x SRF 400)] x EMERALD
I-For*	AGS 457 × SKAF 148	Brown	Green	Forward cross
I-Rec	SKAF 148 × AGS 457	Yellow	Green	Reciprocal cross
II-For	AGS 346 × SKAF 148	Green	Green	Forward cross
II-Rec	SKAF 148 × AGS 346	Yellow	Green	Reciprocal cross

^*^For Forward cross, *Rec* Reciprocal cross

### Crossing program

The genotypes AGS457, AGS346 and SKAF148 were grown in the controlled environmental conditions of the National Phytotron Facility, IARI, New Delhi. The AGS457 and AGS346 were crossed with SKAF148 both in direct and reciprocal ways and the seeds were harvested (Table 1). The F_1_ plants were subjected to hybridity testing and the F_2_ seeds were harvested only from the true F_1_ plants. The F_2_ seeds were grown in the field during 2021 under proper spacing and management and the fully matured F_2:3_ seeds were harvested separately and evaluated for seed coat color inheritance. Similarly, the seed color of the F_3:4_ seeds were also studied to establish the inheritance pattern and the proposed genotypes.

### Seed coat phenotyping

For effective phenotyping of the seed coats, the seeds were harvested when the plants attained full maturity and the pods dried fully (R8 stage) [5]. Seed coat is a maternal tissue; therefore, the phenotype of the seed coat is determined by genotype of the maternal parent where it is produced. As such, the phenotype of seed coat always remains one generation behind the seed generation. This indicates that the phenotype of the seed coat in an F_1_ seed is determined solely by the phenotype of the maternal parent, while the phenotype of the seed coat of the F_2_ seeds will represent the phenotype of the F_1_ generation seeds, and so on. Following this procedure, the seed coat color of each seed was noted for each of the four populations in each of the F_1_, F_2_, F_3_ and F_4_ generations. The F_3_ seeds of each F_2_ plant were classified based on seed coat color (Green, Brown and Yellow) and chi-square test was performed to test goodness-of-fit to the expected ratio. The software R was used to run all χ2 tests [16].

## Results

**Fig. 1:**
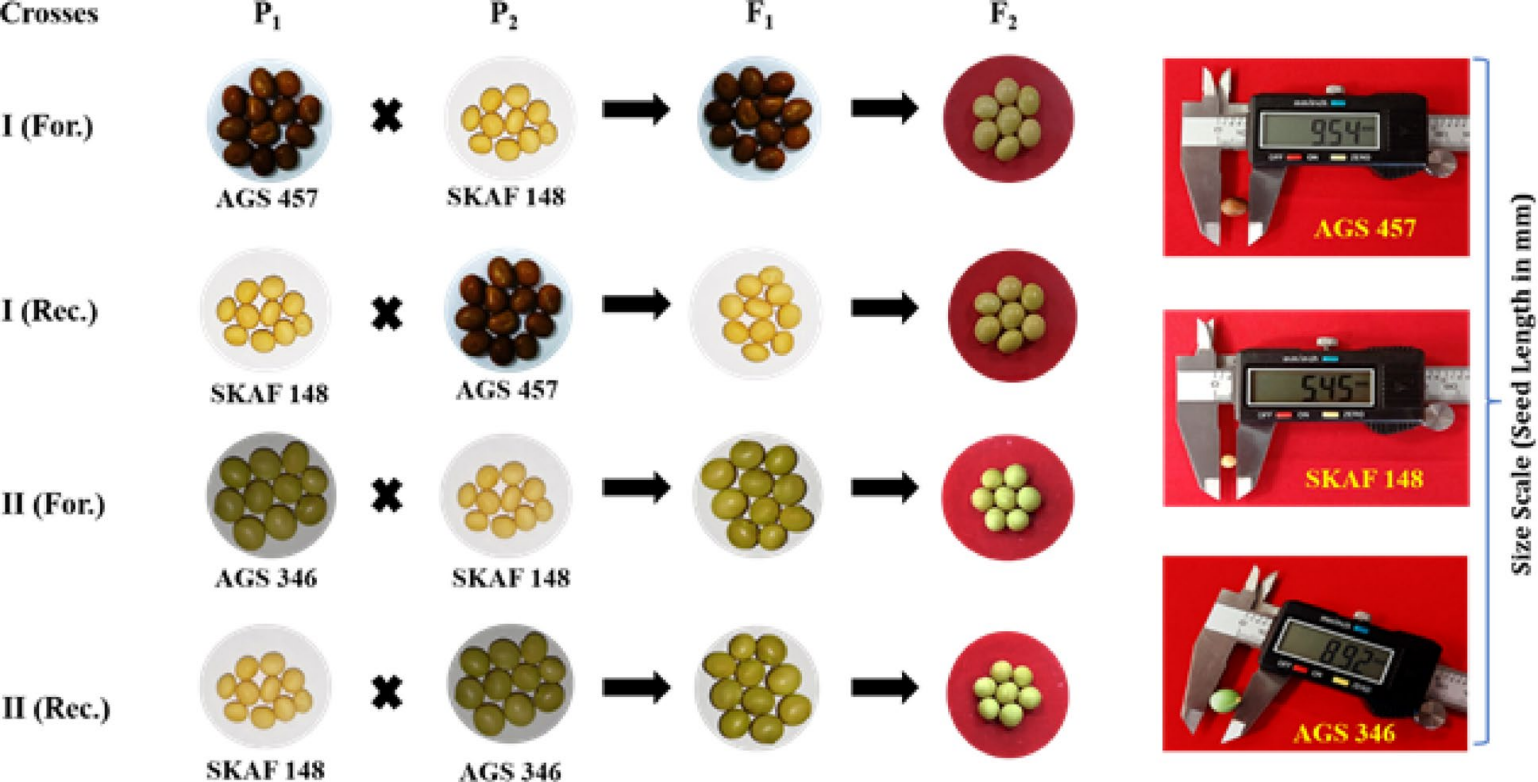
Seed coat colour of parents, F_1_ and F_2_ generations

The genotypes AGS457, AGS346, and SKAF148 were cultivated under controlled conditions at the National Phytotron Facility, IARI, New Delhi. Crosses were performed between AGS457 and SKAF148, as well as between AGS346 and SKAF148, in both direct and reciprocal directions, and the resulting seeds were collected (Table 1). In all the four cross combinations, the seed coat color of the F_1_ seeds were as like the seed coat color of the maternal parents (Fig. 1). The seed coat of the F_2_ seeds were all green, which segregated in to brown, yellow and green seed coat in the subsequent generations. Detail of inheritance of seed coat is presented below as per cross combinations.

### Cross I (Forward)- AGS 457 × SKAF 148

**Fig. 2:**
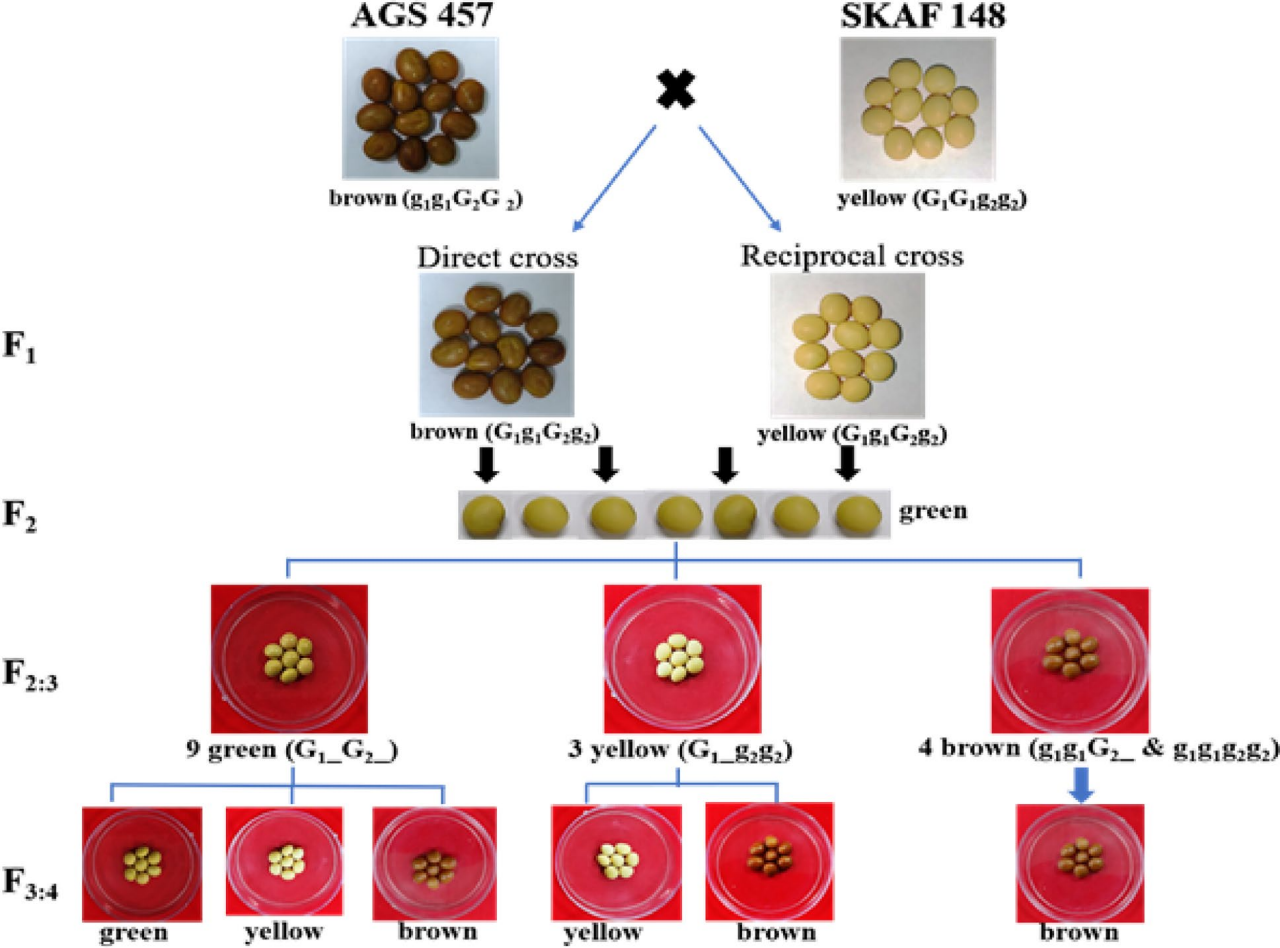
Segregation of seed coat color in different filial generations of cross between AGS 457 and SKAF 148

In this cross, seed coat of the F_1_ seeds was brown, resembling the female parent AGS 457. However, the seed coat of all the F_2_ seeds (237 nos.) was green (Table 1, Fig. 2). The F_3_ seeds produced from the 237 F_2_ plants found to segregate for the seed coat colours as green (147 Nos.), yellow (42 Nos.) and brown (48 Nos.), which fitted well to the 9:3:4 ratio (χ2 = 3.68, *P* = 0.16) indicating a digenic interaction in expression of these seed coat colours (Table 2; Fig. 1). In the F_3_ generation, the 147 green seeds produced plants that segregated for seed coat color. Out of 147 F_3_ plants, 17 plants had only green seeds, 38 plants had green and yellow seeds in 3:1 ratio, 34 plants had green and brown seeds in 3:1 ratio, and 58 plants had green, yellow and brown seeds in 9:4:3 ratio (Table 3). Similarly, the 42 yellow F_3_ seeds germinated into 42 plants, of which 13 produced only yellow seeds and 29 produced yellow and brown seeds in the ratio of 3:1. The 48 brown F3 seeds germinated into 48 plants, which did not show any segregation for coat colour and produced only brown seeds. (Table 3).

**Table 2:** Segregation of seed coat colour in the F_3_ seeds in AGS 457 and SKAF 148 in direct and reciprocal crosses and the proposed genotype of the segregating plants

Crosses	Particular	No. of F_2_ plants with various seed coat colour and proposed genotype			Total F_2_ plants (Nos)	Expected F_2_ ratio	χ^2^ value	*P* value
		Green	Yellow	Brown				
AGS 457 xSKAF 148	Observed Nos	147	42	48	237	9:3:4	3.68	0.16
	Proposed genotype	*G1_G2_*	*G1_g2g2*	*g1g1G2_; g1g1g2g2*				
SKAF 148 × AGS457	Observed Nos	129	54	63	246	9:3:4	2.02	0.36
	Proposed genotype	*G1_G2_*	*G1_g2g2*	*g1g1G2_; g1g1g2g2*

**Table 3:** Segregation for seed coat colour in F_4_ seeds of the cross AGS 457 × SKAF 148

Cross	F_3_ seed coat colour and the proposed genotype	F_3_ plants (No.)	F_4_ seeds (No.)	F_4_ seed coat color			Expected ratio	χ^2^ value	*P* value
				Green	Yellow	Brown			
AGS 457 xSKAF 148	Green (*G1G1G2G2*)	17	176	176	–	–	–	–	–
	Green (*G1G1G2g2*)	38	378	270	108	–	3:1	2.57	0.11
	Green (*G1g1G2G2*)	34	352	256		96	3:1	0.97	0.32
	Green (*G1g1G2g2*)	58	582	328	110	144	9:3:4	0.02	0.98
	Total	147							
	Yellow (*G1G1g2g2*)	13	158	–	158	–	–	–	–
	Yellow (*G1g1g2g2*)	29	328	–	238	90	3:1	1.04	0.31
	Total	42							
	Brown (*g1g1G2G2*, *g1g1G2g2, g1g1g2g2*)	48	572	–	–	572	–	–	–

### Cross I (Reciprocal)- SKAF 148 × AGS 457

The F_1_ seeds produced from this cross found to have yellow seed coat similar to the female parent SKAF148 (Table 1, Fig. 2). The 246 F_2_ seeds, on the other hand, all had green seed coat matching neither of the parental genotypes, which indicated interaction effect of the genes controlling the inheritance of the seed coat color (Table 1). The F_3_ seeds, however, found to segregate for seed coat color. Out of the 246 F_2_ plants, 129 produced seeds with green seed coat, 54 had yellow seed coat, and 63 had brown seed coat fitting perfectly to the 9:3:4 ratio of digenic interaction (χ2 = 2.02, *P* = 0.36) (Table 2). In the F_3_ generation, the plants produced from the 129 green seeds segregated for the seed coat color. Out of 129 F_3_ plants, 14 plants produced only green seeds, 30 plants produced green and yellow seeds in the ratio of 3:1, 34 plants produced green and brown seeds in the ratio of 3:1 and 51 plants produced green, yellow and brown seeds in the ratio of 9:3:4. Similarly, the 54 yellow F_3_ seeds germinated into 54 plants, of which 17 produced only yellow seeds and 37 produced yellow and brown seeds in the ratio of 3:1. The 63 brown F_3_ seeds produced plants that had only brown seeds without any segregation (Table 4).

**Table 4:** Segregation for seed coat color in F_4_ seeds of the cross SKAF 148 × AGS 457

Cross	F_3_ seed coat color and the proposed genotype	F_3_ plants(Nos.)	F_4_ seeds(Nos.)	Nos. of F_4_ seeds with various seed coat color			Exp. ratio	χ2 value	*P* value
				Green	Yellow	Brown			
SKAF 148 × AGS 457	Green (*G1G1G2G2*)	14	154	154	–	–	–	–	–
	Green (*G1G1G2g2*)	30	330	258	72	–	3:1	1.78	0.18
	Green (*G1g1G2G2*)	34	342	255	–	87	3:1	0.04	0.85
	Green (*G1g1G2g2*)	51	556	306	92	158	9:3:4	4.18	0.12
	Total	129							
	Yellow (*G1G1g2g2*)	17	138	–	138	–	–	–	–
	Yellow (*G1g1g2g2*)	37	294	–	210	84	3:1	2.00	0.15
	Total	54							
	Brown (*g1g1G2G2, g1g1G2g2, g1g1g2g2*)	63	518	–	–	518	–	–	–

The genotype proposed for various phenotypes in the forward cross of this combination holds true for it as well. The segregation of the alleles and the corresponding phenotypes also found to align with it (Table 4). It thus confirmed the digenic model with recessive epistasis in the development of the seed coat colour in the tested soybean lines.

### Cross II-forward (AGS 346 × SKAF 148)

**Fig. 3:**
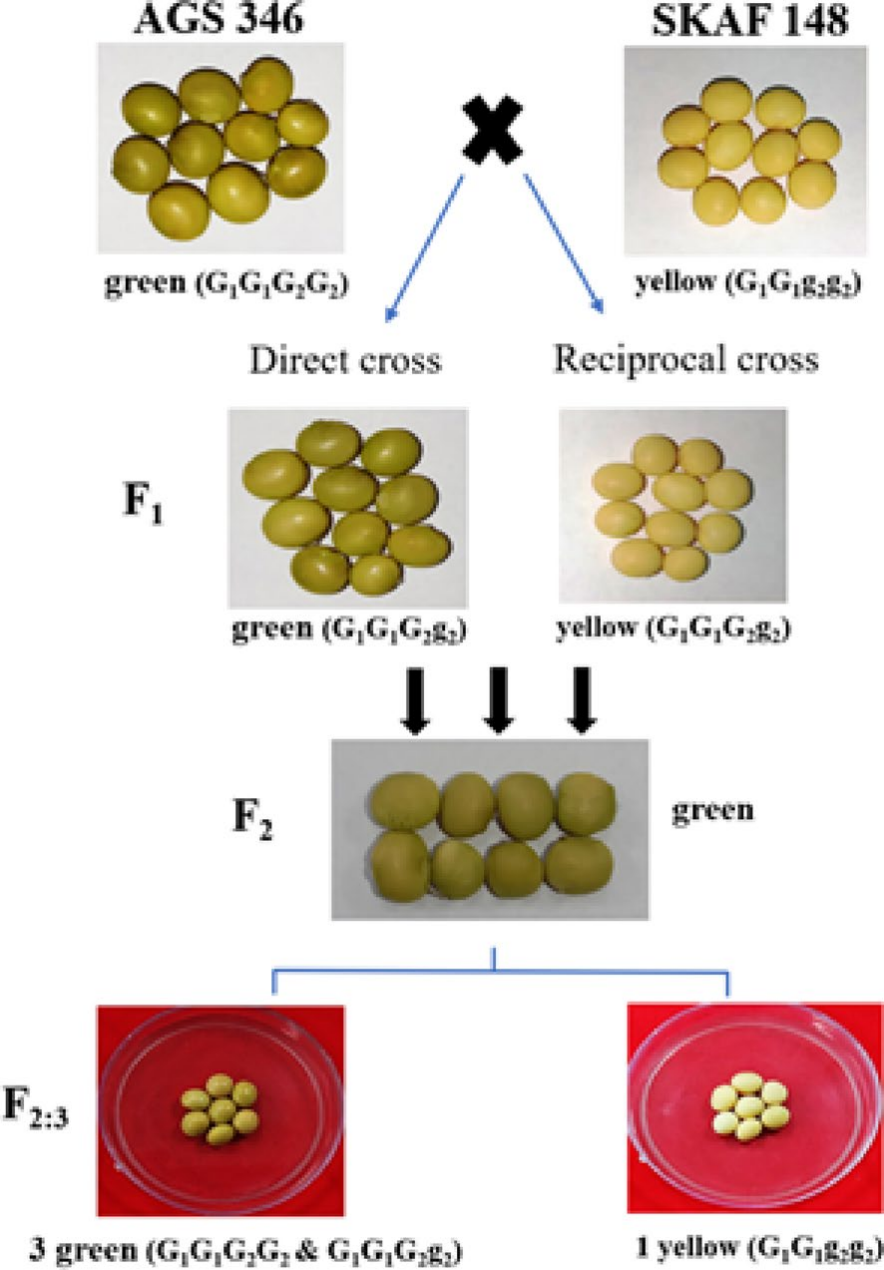
Segregation of seed coat color in different filial generations of cross between AGS 346 and SKAF 148

When green-seeded AGS346 was crossed with yellow-seeded SKAF148, all the F_1_ seeds had a green seed coat, resembling the female parent AGS 346 (Table 1, Fig. 3). The 291 F_2_ seeds all had green seed coat (Table 1). However, the F_3_ seeds found to segregate for the seed coat colour. Out of 291 F_2_ plants, 210 and 81 plants had green and yellow seeds in the ratio of 3:1, respectively (χ2 = 1.25, *P* = 0.26).) (Table 5). In the F_3_ generation, the yellow seeds did not segregate and produced only yellow seeds while the green seeds again segregated to produce green and yellow seeds in the ratio of 3:1 (Fig. 3). In this cross also, the F_2_ seeds having F_1_ seed coat were green confirming the interaction between the genes in production of the seed coat colour. The segregation pattern of seed coat colour in the F_3_ generation supported the digenic model of gene interaction in controlling the seed coat colour.

**Table 5:** Segregation of seed coat colour in the F_3_ seeds in AGS 346 and SKAF 148 in direct and reciprocal crosses and the proposed genotype of the segregating plants

Crosses	Particular	No. of F_2_ plants with various seed coat colour and proposed genotype			Total F_2_ plants (Nos)	Expected F_2_ ratio	χ^2^ Value	*P* value
		Green	Yellow	Brown				
AGS346 x SKAF 148	Observed Nos	210	81	–	291	3:1	1.25	0.26
	Proposed genotype	*G1G1G2_*	G1G1g2g2	–				
SKAF 148 × AGS346	Observed Nos	194	55	–	249	3:1	1.13	0.29
	Proposed genotype	*G1G1G2_*	G1G1g2g2	–				

### Cross II-reciprocal (SKAF 148 × AGS 346)

The F_1_ seeds obtained from this cross had yellow seed coat like the maternal parent SKAF148 (Table 1, Fig. 3). However, the 249 F_2_ seeds obtained from the F_1_ plants all had green seed coat (Table 1). It was same as observed in its forward cross i.e. AGS346 x SKAF148. Out of the 249 F_2_ plants, 194 plants produced green seeds and the rest 55 produced yellow seeds, which was in 3:1 ratio (χ2 = 1.13, *P* = 0.29) (Table 5). In the F_3_ generation, the 194 green seeds produced plants with green and yellow seeds in the ratio of 3:1 while the 55 yellow seeds produced plants with yellow seeds only (Fig. 3). The inheritance pattern in the direct and reverse cross of AGS346 x SKAF148 also supports the theory of digenic involvement in production of seed coat colour through recessive epistasis.

### Proposed genotypes

Based on the segregation pattern of seed coat colors in the 4 cross combinations across the generations, we propose that two genes, *G1* and *G2*, each with two alleles- *G1, g1* and *G2, g2,* determine the seed coat color. Plants with both *G1* and *G2* genes, either in dominant homozygous (*G1G1G2G2*) or heterozygous (*G1g1G2g2*) conditions, produce green seeds (like AGS346). In the absence of dominant G2, the G1 in either homozygous (*G1G1*) or heterozygous (*G1g1*) state results in yellow seeds. However, when G1 is recessive homozygous (*g1g1*), it suppresses the expression of G2, leading to brown seeds. Based on this, the genotypes of the parental lines are proposed as *g1g1G2G2* for AGS457 (brown seed coat), *G1G1g2g2* for SKAF148 (yellow seed coat) and *G1G1G2G2* for AGS346 (green seed coat) (Table 1).

Since the seed coat is maternal tissue, the F_1_ seeds inherit the seed coat color from the female parent. However, the interaction of both G1 and G2 genes in the F_1_ plants (though not expressed in the seed coat) would produce green seeds in the F_2_ generation, reflecting the F_1'_s heterozygous genotype (Table 1). The F_3_ seeds (with the F_2_ seed coat), however, segregate for green, yellow, and brown in a 9:3:4 ratio, indicating recessive epistasis between G1 and G2 genes. This proposed ratio is supported by the chi-square test (Table 2). Similarly, the F_4_ seed coat color also segregates according to the proposed genotypes for each class, which is confirmed by the chi-square test (Table 3).

## Discussion

In soybean, seed coat color has undergone a fascinating evolution, transitioning from black in wild soybean (*Glycine soja*) to a vibrant spectrum in domesticated varieties (*Glycine max*) [10]. Due to its simplicity, seed coat color remains a valuable phenotypic marker in soybean breeding [6, 7]. However, the underlying genetics defy easy explanation. Despite the identification of several genetic loci and their inheritance patterns, a clear picture remains elusive. Terao [21] pinpointed a single factor (G1) responsible for green seed coats in offspring with yellow mothers, but observed no segregation when the mother was green, resulting in F_1_ and F_2_ generations mirroring the maternal color. Terao's findings were corroborated by Piper and Morse [15], who proposed two sorts of green responsible for these puzzling outcomes. Evidently, one form of green exhibited maternal inheritance, while the other followed Mendelian principles. Similarly, a handful of studies [25], [13], [23] reported d1 and d2 loci controlling the green seed embryo in Columbia and T104 genotypes. Reese and Boerma [17] further identified G2 and g3 genes governing green seed coat color, outlining their Mendelian inheritance pattern. Driven by these complexities, this study delves into the systematic inheritance of seed coat color and aims to identify the governing loci in soybean.

Brown (AGS 457) and yellow (SKAF 148) soybeans were crossed in both direct and reciprocal ways to see how the seed coat color is inherited. Interestingly, the seed coat color of the F_1_ offspring depended on which parent was its mother. This aligns with findings by Chandlee and Vodkin [4]. The seed coat forms from the mother plant's tissues, not the embryo inside. So, the seed coat color reflects the mother's genes (genotype), not the genes of the seed itself. This explains why F_2_ seeds from both crosses were green: they inherited two dominant genes (*G1_G2_*) from the F_1_ mother, regardless of the pollen source. In both forward and reciprocal crosses, the F_2_ generation showed a mix of green, yellow, and brown seed coats in the 9:3:4 ratio. It suggests recessive epistasis interaction of two genes in controlling the seed coat color. Green seeds (*G1_G2_*) come from a combination of brown (*g*_*1*_*g*_*1*_*G*_*2*_*G*_*2*_) and yellow (*G1G1g2g2*) genes.

Bhatt and Torrie [2] crossed brown (T25) and yellow soybeans (Harasoy) and got the segregation pattern of yellow, brown, and buff in the ratios of 12:3:1. Song et al. [19] used the cross of the yellow seed coat (ZP95-5383) and brown seed coat genotype (NY279) to determine the segregation pattern of the green, yellow, brown, and black seed coats. However, our study for the first time has shown that the seed coat color in soybeans is controlled by two genes in a recessive epistasis interaction. To confirm this, we advanced the seeds to F_3_ and F_4_ generations and looked at the seed coat colors. As expected, the homozygous dominant (*G1G1G2G2*) families with green seed coat did not segregate and remained green in the later generations too. Other combinations of genes resulted in different color patterns. While a heterozygous family with the genetic makeup *G*_*1*_*g*_*1*_*G*_*2*_*G*_*2*_ segregated for green and brown offspring, a heterozygous family with genotype *G*_*1*_*G*_*1*_*G*_*2*_*g*_*2*_ segregated for green and yellow offspring. Similarly, the heterozygous plants with genotype *G*_*1*_*g*_*1*_*G*_*2*_*g*_*2*_ segregated for green, yellow, and brown progenies in the ratio of 9:3:4. The family with a genetic constitution *G*_*1*_*g*_*1*_*g*_*2*_*g*_*2*_ segregated into yellow and brown, the yellow seed coat family (*G1G1g2g2*) did not segregate. The families with brown seed coat (*g*_*1*_*g*_*1*_*G*_*2*_*_* and *g*_*1*_*g*_*1*_*g*_*2*_*g*_*2*_) did not segregated. All these patterns of inheritance happened as expected from the proposed genotypes, which further supported our idea of two interacting genes controlling seed coat color in soybean.

When the genotypes with green seed coat i.e. AGS 346 (*G*_*1*_*G*_*1*_*G*_*2*_*G*_*2*_) and yellow seed coat SKAF 148 (*G*_*1*_*G*_*1*_*g*_*2*_*g*_*2*_) were crossed, the F_1_ seeds were green, while the seeds of its reciprocal cross were yellow. This happened as expected from the maternal effect. The F_2_ seeds, on the other hand, were green in both the crosses, because the seed coat of the F_2_ seeds were of the F_1_ type (*G1G1G2g2*). Interaction of both the dominant alleles i.e. G1 and G2 produced the green seed coat. In F_2:3_ generation (Seed coat is F_2_), the heterozygous allele i.e. *G2g2* segregated and produced green and yellow seed coat in 3:1 ratio in both direct and indirect crosses. This is also as expected from the proposed genotype and gene interaction. Previous studies ([21], [2], [17]) reported a single gene model for the green and yellow seed coats, with the green seed coat being dominant over yellow. We too found the green color to be dominant over yellow; however, unlike their single gene model, we found two genes to interact to produce the green colour. The G genes (including G1 and G2), contribute to green pigmentation by reducing chlorophyll degradation, a characteristic associated with the “stay-green” phenotype observed in soybean [19].

It was found that the base colour of the seed coat was brown, which got converted to green with yellow as its intermediate product i.e. brown → yellow → green. The product of the G1 gene catalysed conversion of brown to yellow, which was then catalysed by the product of the gene G2 to produce green seed coat. In the absence of G1 alone (i.e. *g1g1 G2_*) or G1 and G2 together (i.e. *g1g1g2g2*), the colour remained brown. The catalytic reaction of G1 converted brown to yellow; however, it remains so in the absence of G2 (i.e. *G1_g2g2*). It is, however, to be noted that in soybean, there are black seed coat, as well, primarily in the wild types [11]. Therefore, it would be interesting to investigate its relationship with other colour such as brown, green and yellow.

## Conclusion

The inheritance pattern of seed coat color in two soybean crosses, AGS 457 × SKAF 148 and AGS 346 × SKAF 148 (both forward and reciprocal), revealed a digenic interaction effect, meaning that two genes jointly determined the trait. The genotype *g1g1G2G2* encoded brown seed coat (AGS457) and *G1G1g2g2* encoded yellow seed coat (SKAF148), while the combination of these brown and yellow seed coat genes (*G1_G2_*) produced green seed coats (AGS346). These observations suggested a recessive epistasis, where the homozygous recessive alleles of one gene (*g1g1*) masked the effect of the dominant alleles of another gene (*G2G2*), resulting in a brown seed coat. Understanding the maternal effect and recessive epistasis in seed coat colour inheritance provides valuable insights that can be directly applied by soybean breeders to develop new varieties with desired seed coat colours.

## Data Availability

All relevant data supporting the findings of this study are available in this article.
